# Perturbation Free-Energy Toolkit: An Automated Alchemical Topology Builder

**DOI:** 10.1021/acs.jcim.1c00428

**Published:** 2021-08-20

**Authors:** Drazen Petrov

**Affiliations:** Department of Material Sciences and Process Engineering, Institute of Molecular Modeling and Simulation, University of Natural Resources and Life Sciences Vienna, Muthgasse 18, A-1190 Vienna, Austria

## Abstract

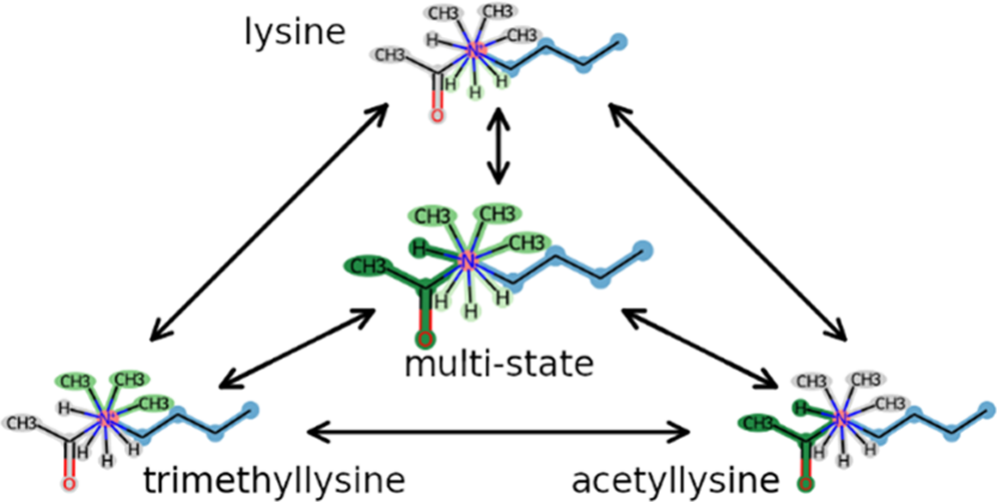

Free-energy calculations play an important role in the application of computational chemistry to a range of fields, including protein biochemistry, rational drug design, or materials science. Importantly, the free-energy difference is directly related to experimentally measurable quantities such as partition and adsorption coefficients, water activity, and binding affinities. Among several techniques aimed at predicting free-energy differences, perturbation approaches, involving the alchemical transformation of one molecule into another through intermediate states, stand out as rigorous methods based on statistical mechanics. However, despite the importance of free-energy calculations, the applicability of the perturbation approaches is still largely impeded by a number of challenges, including the definition of the perturbation path, i.e., alchemical changes leading to the transformation of one molecule to the other. To address this, an automatic perturbation topology builder based on a graph-matching algorithm is developed, which can identify the maximum common substructure (MCS) of two or multiple molecules and provide the perturbation topologies suitable for free-energy calculations using the GROMOS and the GROMACS simulation packages. Various MCS search options are presented leading to alternative definitions of the perturbation pathway. Moreover, perturbation topologies generated using the default multistate MCS search are used to calculate the changes in free energy between lysine and its two post-translational modifications, 3-methyllysine and acetyllysine. The pairwise free-energy calculations performed on this test system led to a cycle closure of 0.5 ± 0.3 and 0.2 ± 0.2 kJ mol^–1^, with GROMOS and GROMACS simulation packages, respectively. The same relative free energies between the three states are obtained by employing the enveloping distribution sampling (EDS) approach when compared to the pairwise perturbations. Importantly, this toolkit is made available online as an open-source Python package (https://github.com/drazen-petrov/SMArt).

## Introduction

Calculation of free-energy differences is one of the main objectives in computational chemistry as such differences characterize chemical processes, directly determining properties such as ligand binding affinities or partition coefficients. Perturbation free-energy calculations, involving the alchemical transformation of one chemical into another via a pathway of unphysical intermediate states, present a rigorous approach derived from statistical mechanics.^1−12^ Several such methods have been developed over the years, including, for instance, thermodynamic integration,^13^ its extended version,^14^ or Bennett’s acceptance ratio.^15^ More recently, nonequilibrium techniques like the Crooks Gaussian intersection method^16,17^ and the Jarzynski equality^18,19^ have also been applied. While more tractable than the direct simulations of the actual physical process (e.g., ligand binding), perturbation simulations are still computationally demanding, presenting one of the major impediments of their wider application.

The efficiency of different perturbation methods in various contexts has been studied.^17,20−23^ In addition, the effects of the choice of intermediate states and exact coupling of the transformation to the Hamiltonian of the system through a coupling parameter λ have been explored.^24−28^ Related to this, the transformation pathway depends on the definition of alchemical changes, which, in turn, might strongly affect the performance of the calculations. In particular, the dual topology approach either replaces (by perturbing into and from noninteracting dummy atoms) all atoms of one compound with the atoms of the other^29,30^ or replaces only a subset of nonmatching atoms while keeping atoms with matching atom types unperturbed.^31,32^ Alternatively, only a subset of nonmatching atoms can be perturbed into each other while minimizing the number of such perturbations, i.e., the single topology approach,^29,31,32^ which is especially beneficial when compounds in the question share the same scaffold. Performing free-energy calculations using such an approach usually involves a cumbersome and often manual procedure of defining the perturbations, choosing intermediate states and the amount of sampling for simulations, followed by analysis of the collected data. On the other hand, several available tools allow for automatization of some of the steps involved in the process, including the generation of perturbation topologies.^30,32−38^ To name a few examples: pmx provides^34,36^ a database of amino-acid building blocks aimed at perturbation free energy calculations of point mutations and modifications based on the single topology strategy and the GROMACS simulation package; ProtoCaller^38^ combines several open-source packages where a modified RDKit^39^ MCS algorithm is used to generate perturbation topologies, the while FESetup^35^ aims at the preparation and postanalysis of free-energy calculations using multiple MD engines.

In this study, an automated perturbation topology builder based on a graph-matching algorithm was developed allowing the user to find the maximum common substructure (MCS) of two or a set of multiple compounds and define the perturbation accordingly. Several MCS search options will be presented, leading to alternative definitions of perturbation pathways for a diverse set of compounds and perturbation problems, ranging from simple example systems to sets of multiple ligands. Additionally, this tool was used to generate perturbation topologies and to calculate the free-energy differences between lysine and two of its post-translational modifications. Finally, the toolkit is made available as an open-source Python package via a GitHub repository (https://github.com/drazen-petrov/SMArt).

## Methods

### Perturbation Topology Builder

**Figure 1:**
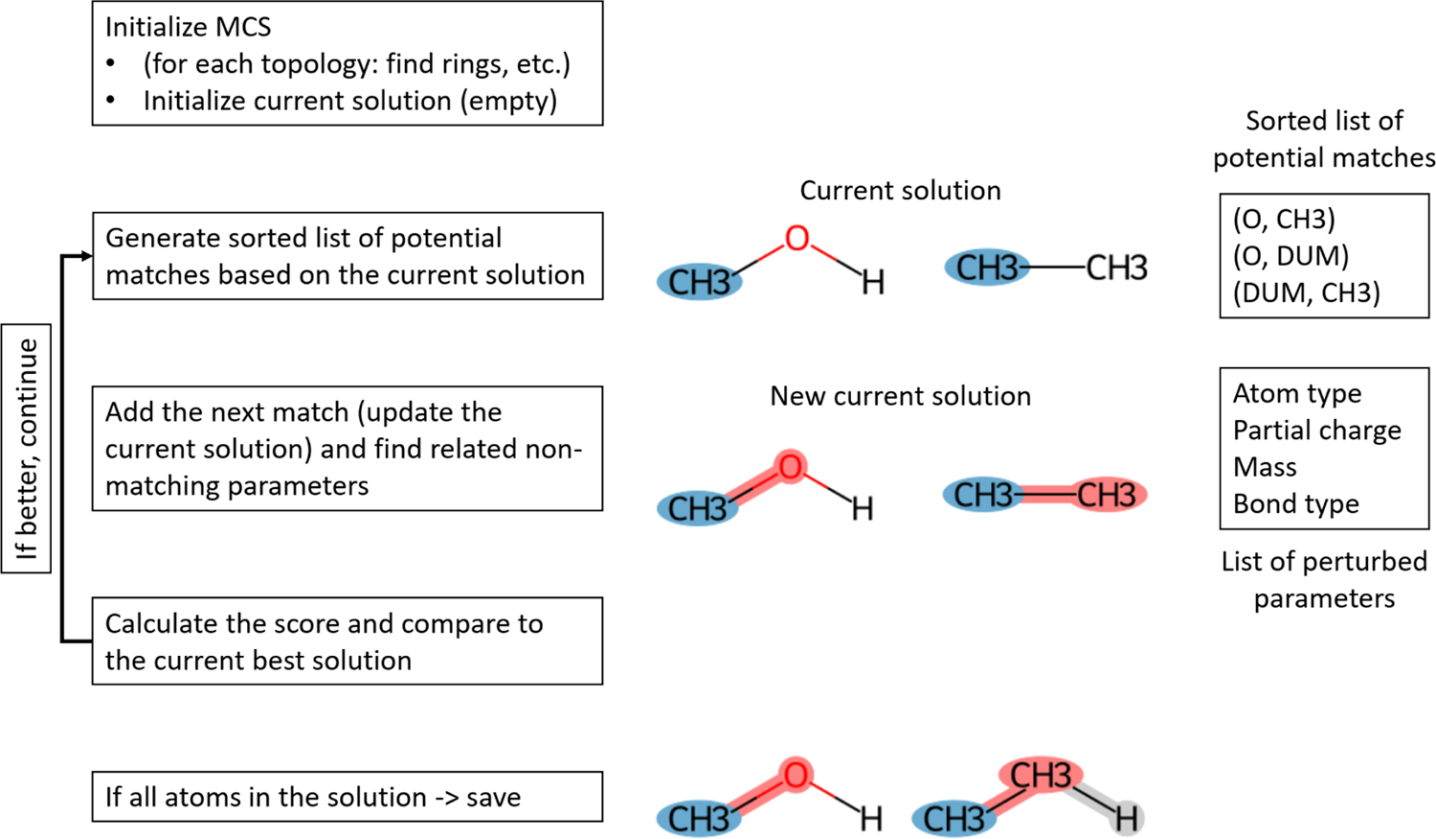
General workflow of the perturbation topology builder. Algorithm steps are shown on the left side. Step (0) initializes all relevant attributes and parameters needed for the search and performs a simple analysis of the input topologies (e.g., finding rings). After the initialization step, an iterative search procedure is initiated. Step (1) based on the current solution (in the very first call, empty solution, i.e., no matched atoms) generates a list of all possible and allowed atom matches (pairs) and sorts them according to the estimated number of additional atom matches that are possible after the atom match in the question is added to the solution (related to the score calculated in step 3). Step (2) adds the next atom pair (match) from the sorted list from step 1 to the current solution and updates it. Step (3) calculates a score based on the user-defined function and compares it to the best score (the best solution) enumerated thus far in the execution of the algorithm. If the score is better than the one of the current best, the algorithm continues with a next iteration of step 1. If all atoms of all input topologies are present in the solution, the solution is saved. An illustration of the execution of the algorithm is shown on the right based on a toy perturbation problem between methanol and ethane (compounds represented according to the GROMOS force field where the methyl groups are modeled as a single united-atom particle). The current solution in step 1 with two methyl groups matched (in blue) is shown. Accordingly, the list of potential next atom matches is generated based on the first neighbors of the atoms in the current solution. Note that matches with dummy atoms are also explicitly included in the algorithm. The updated current solution after adding the next atom match between the oxygen and the second methyl in step 2 is shown (highlighted in red to notify perturbation in atom type, partial charge, and mass, with perturbed bonds also marked in red). Finally, the solution with the maximum number of matched atoms is shown at the bottom, where the hydrogen atom in the methanol state is marked for removal, i.e., perturbation into a noninteracting dummy atom and the other way around in the ethane state (dummy atom highlighted in light gray).

The perturbation topology builder presented in this study uses the single topology approach to create a definition of the perturbation pathway for a set of input molecular topologies (at least two), needed for free-energy calculations based on the maximal common substructure. The maximal common substructure (MCS) search for the two molecules involved in the perturbation is based on the VF algorithm for graph isomorphism matching.^40^ It involves an iterative procedure (Figure 1), in which in each step, a pair of atoms, each belonging to one of the compounds, is added to the current common substructure (current solution).

At the beginning of each iteration, the list of available pairs of atoms to be added in the current solution is updated based on the first neighbors of the atoms in the current solution. Upon adding a pair of atoms, the common part of molecular topologies is checked for nonmatching force-field parameters. For instance, matching the oxygen in methanol to the carbon/methyl in ethane leads to several nonmatching nonbonded and bonded parameters, including the mass, the atom type, partial charge, and the bond type. The nonmatching force-field parameters as well as the potential introduction of dummy atoms contribute to a score based on user-defined penalty. A crucial part of this update is an estimate of the minimal penalization score that this current solution can achieve, according to which the list of available pairs of atoms is sorted. This ensures that solutions with low penalty scores are found early in the enumeration. When a current solution’s minimal possible score is higher than a score of an already enumerated solution, this branch of enumeration is pruned. An initial point in the algorithm is a list of all available pairs of atoms, equaling *n* × *m*, where *n* and *m* stand for the number of atoms in each of the compounds.

The algorithm can also be simultaneously applied on a set of multiple topologies, where the resulting match represents the minimum structure of which each individual compound is a substructure, or simply put, a common scaffold. This can be used to perform enveloping distribution sampling (EDS)^41−43^ or generate closed thermodynamic cycles on a set of multiple compounds.

The algorithm is implemented in the Python programming language and supports GROMOS and GROMACS file formats. Package documentation, including a tutorial with an example code was generated using the Sphinx tool and Jupyter Notebooks.^44^ Illustrations of different perturbation pathways were generated using the RDKit package.^39^

### Perturbation Simulations

Molecular dynamics simulations were performed using the GROMOS11^45^ and GROMACS^46^ simulation packages. The united-atom GROMOS force-field parameter set 54A8,^47−49^ SPC explicit water,^50^ and a 2 fs integration step were used. The temperature and the pressure were kept constant at 300 K and 1 bar using weak coupling with a relaxation time of 0.1 and 0.5 ps, respectively.^51^ Pressure scaling was applied isotropically, with an isothermal compressibility of 4.575 × 10^–4^ (kJ mol^–1^ nm^–3^)^−1^. A reaction-field contribution was added to the electrostatic interactions and forces to account for a homogeneous medium with a dielectric permittivity of 61 outside the cutoff sphere. In simulations using the GROMOS11 molecular simulation package, a molecular pair list was generated using a triple-range cutoff,^52^ where nonbonded interactions up to a short range of 0.8 nm were calculated at every time step from a pair list that was updated every 5 steps. Interactions up to a long-range cutoff of 1.4 nm were calculated at pair list updates and kept constant in between. The SHAKE algorithm was used to constrain the bond lengths to their optimal values with a relative geometric accuracy of 10^–4^.^53^ In simulations performed using the GROMACS simulation package, the Verlet pair-list algorithm^54,55^ was used together with the LINCS algorithm^56^ for constraining the bond lengths to their optimal values. Coordinates and energies were saved every 50 ps.

The above-described perturbation topology builder and the default multistate MCS search (maximizing the number of matched atoms, while restricting any perturbations in the bonded interactions—aimed for generating EDS topologies) were used to define alchemical perturbations between lysine, 3-methyllysine, and acetyllysine. A soft-core potential was used for perturbations of nonbonded interactions (GROMOS).^57^ Note that a different definition of the soft-core potential is used in GROMACS (see the manual for details). Free-energy changes of these pairwise transformations of a small pentapeptide (GGXGG, where X stands for the affected residue with charge-neutral terminal caps) in the free state, i.e., in water, were performed with the GROMOS and the GROMACS simulation packages. Additionally, the accelerated EDS (A-EDS) approach^43,58^ was used to calculate the relative free energies between the three states, where the calculations were performed with the GROMOS simulation package. Pymol^59^ and the Vienna-PTM webserver^60^ were used to prepare and manipulate PDB files.

Each pairwise perturbation was simulated using 21 equidistant λ-points, with 0.5 ns equilibration time and 5 ns simulation (data collection) time per λ-point. The free-energy differences were calculated using the multistate Bennett acceptance ratio (MBAR).^61^ Two nonequilibrium parameter-search simulations for the A-EDS were performed. First, the A-EDS parameters *E*_min_ and *E*_max_ and the free-energy offset parameters for each of the states were optimized simultaneously for 20 ns. The free-energy offset parameters were further optimized for 20 ns in the second parameter-search simulation while keeping the A-EDS parameters *E*_min_ and *E*_max_ constant (values obtained from the first optimization step). The sigma level of 2 and memory relaxation times of 1 ps (τ_A_) and 2 ns (τ_B_) were used. Subsequently, A-EDS equilibrium sampling simulation was performed for 100 ns, with the A-EDS parameters assigned to the values determined during the parameter-search simulations. The free-energy differences between the states were calculated from the free-energy difference of the states to the A-EDS reference obtained from Zwanzig’s equation.^62^

## Results and Discussion

### Perturbation Topology Builder

An initial step in perturbation free-energy calculations is the definition of the alchemical pathway. An automated tool, based on the single topology approach, able to generate a perturbation topology of two compounds by finding their maximum common substructure was developed. It is based on the VF algorithm for graph isomorphism matching,^40^ where the potential common substructures are enumerated iteratively. A pruning function ensures reasonable running times, even though this is not a guarantee, as graph isomorphism matching is of exponential complexity. This notwithstanding, several tests on small molecules, sets of ligands, post-translational modifications, and amino-acid mutations were completed within seconds. Importantly, while enumerating the substructures, the algorithm also evaluates the perturbations based on molecular topologies, making it possible to guide the search toward different outcomes, via a user-defined score function.

### Score Function

**Figure 2:**
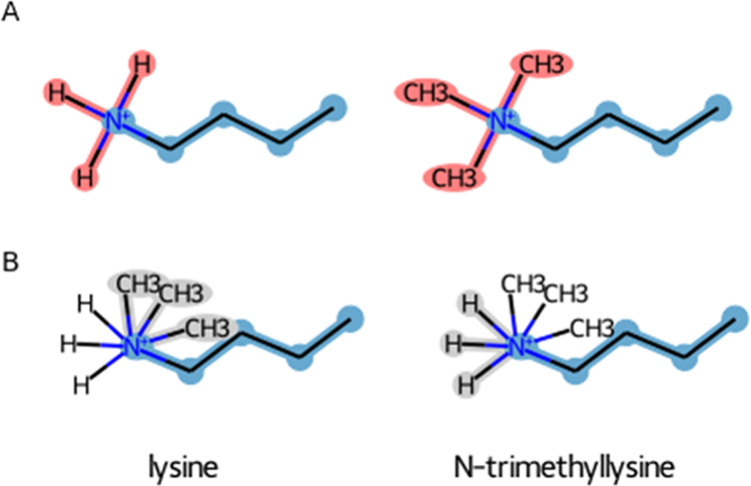
Alternative scenarios for the methylation of the lysine sidechain. The maximum common substructure of lysine (left) and its methylated form (right). (A) Perturbation topologies are generated by maximizing the number of matching atoms and (B) by excluding atom matches that lead to perturbed bonds. Unperturbed atoms are highlighted in blue, perturbed atoms in red, and dummy atoms in gray.

A score is calculated in each iteration step for the current partial solution. It is primarily based on the number of matched atoms, such that the number of matched atom types as defined by the force field is maximized. This is arguably one of the most common choices (also, the default score function), and when performed on lysine trimethylation modification (Figure 2A) results in all atoms being mapped to each other, in which three hydrogen atoms are assigned for perturbation into methyl groups (note that methyl groups within the GROMOS force field are modeled as a single united-atom particle).

Note that the search finds six best solutions with equal scores due to the symmetry. By providing the coordinates of both compounds, the atom-positional root-mean-square deviation (RMSD) based on the matched atoms can be used to distinguish the six solutions and find the best one. While completely irrelevant for this example, this option may be used to optimize the perturbation pathway for ligands bound in a pocket by preferentially selecting matches between atoms that are close in 3D space. This approach can be easily extended to the multistate MCS search, using root-mean-square of pairwise RMSD, i.e., ⟨RMSD^2^⟩^1/2^ or using root-mean-square fluctuations, ⟨RMSF^2^⟩^1/2^. As shown in ref (63), the two quantities are directly related to each other
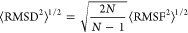
1where *N* is the number of compounds. Both approaches are implemented and can be used in the MCS search.

On the other hand, one can design other matching criteria, for instance, by excluding solutions leading to perturbed bonds, while still maximizing the number of matched atoms, which would lead to a different perturbation topology. In the case of lysine methylation, the hydrogen atoms are perturbed into dummy atoms, while the methyl groups are grown from dummy particles, with the rest of the atoms being matched (Figure 2B). It is worth noting that the algorithm allows for wide flexibility in tuning the MCS search by setting different penalty weights for different types of individual perturbations compared to each other, including atom types, perturbed bonds, angles, proper, and improper dihedrals. The enumerated solutions are sorted according to the score defined by the penalty weights. In addition, this feature can be used not only to select the preference toward a specific type of perturbation but also to generate different perturbation definitions (pathways) between a given pair of compounds of interest, which can be tested for their performance. Practically, this can be done by implementing a general score function and passing it to the MCS search algorithm. Note that these options related to the score (and the score function) are exemplified in a tutorial Jupyter Notebook available within the repository.

### Allowed Atom Matches

**Figure 3:**
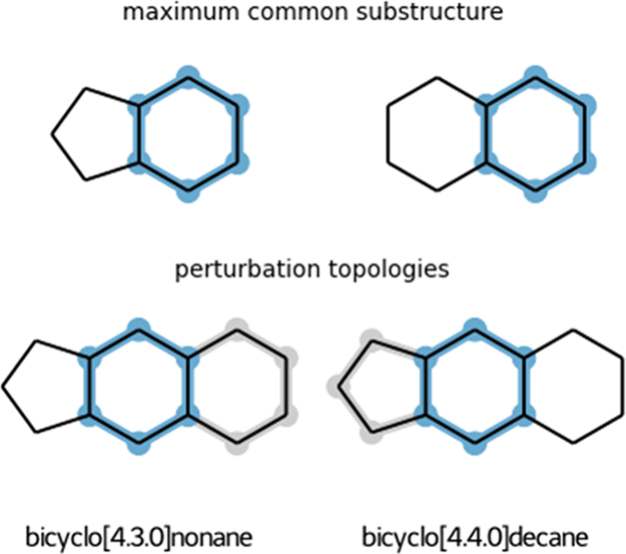
Illustration of the maximum common substructure (highlighted in blue) if the partial ring match is allowed (top row). Perturbation topologies for each of the states (bottom row). Unperturbed atoms are highlighted in blue and dummy atoms in gray.

When it comes to matching ring structures, the algorithm allows for two options: (1) partial match of polycyclic compounds where only a complete match of individual rings is allowed (Figure 3) and (2) only complete match is allowed. Note that only fused rings are taken into account for the partial ring match, while bridged rings are only checked for a complete match. Spiro rings are considered as separate rings for the MCS search.

**Figure 4:**
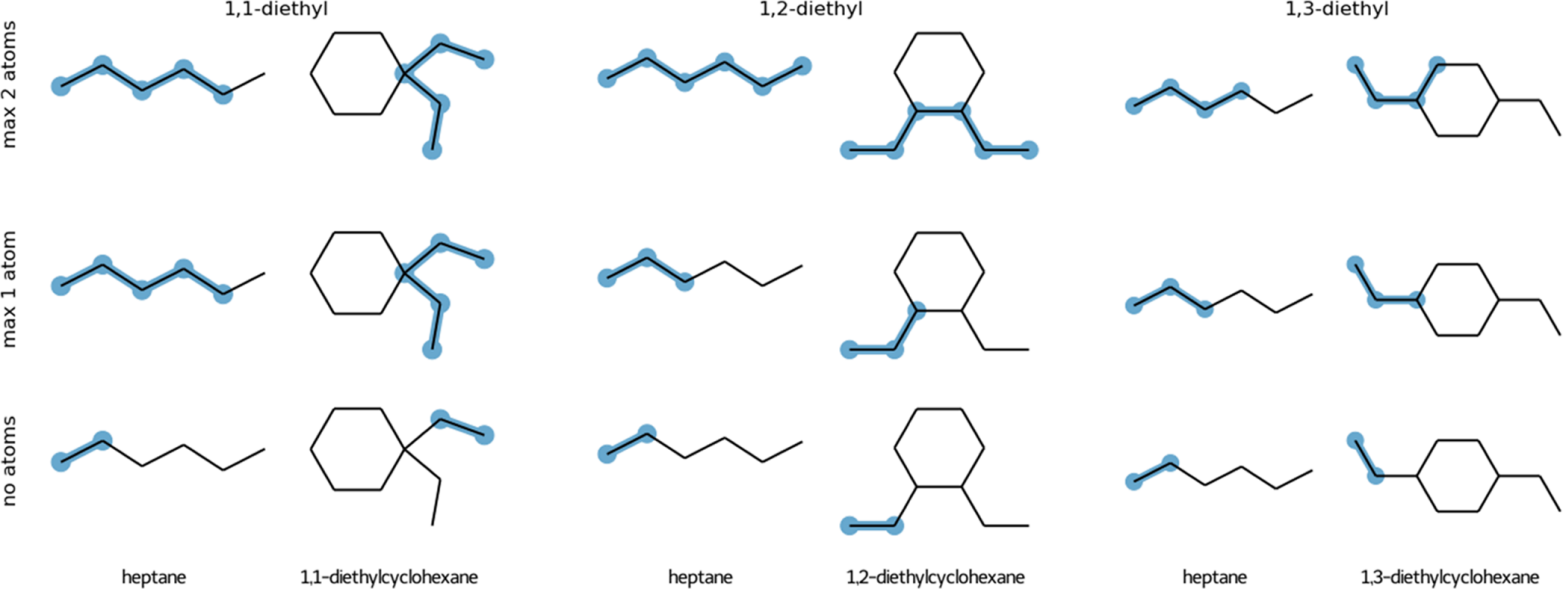
Illustration of the maximum common substructure (highlighted in blue) when matching nonring with ring structures. Top row: partial match of a maximum of two atoms (that share a bond), middle row: partial match of only one atom, and bottom row: no atom match allowed. For simplicity, only the MCS is shown without perturbation topologies that contain additional dummy atoms that are not part of the MCS match.

In the case of matching between ring and nonring atoms, three options are provided: (1) partial match of a maximum of two atoms (that share a bond), (2) partial match of only one atom, and (3) no match of a ring to a nonring atom is allowed (Figure 4). Option 1 was chosen to be default in both cases, as it permits for matching larger maximum common substructure. Note that allowing for a partial match of three or more atoms in a ring structure would potentially affect the sampling of the conformational space of the end states. Arguably, allowing a partial match of two atoms that share a bond would not have such an effect; however, this assumption remains to be tested in simulation. For this reason, alternative choices are provided allowing one or no atom as a partial match. This choice also affects (in the same manner) matching between two rings for which no partial or complete match is found (Figure S1).

### Additional Search Options and Considerations

**Figure 5:**
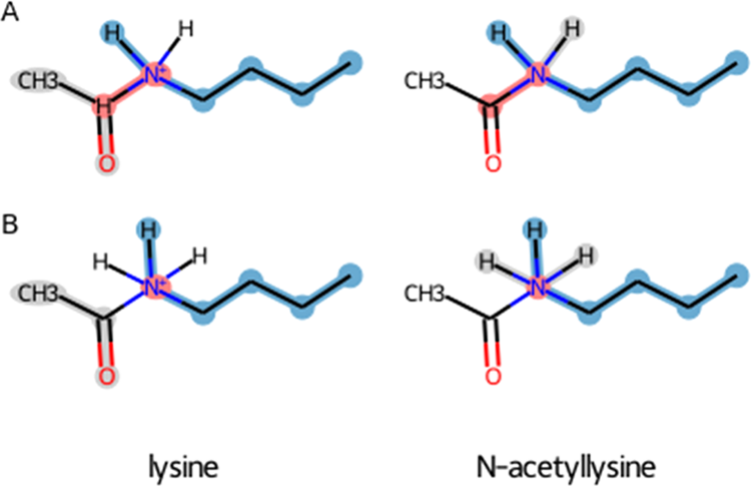
Alternative scenarios for lysine acetylation. The maximum common substructure of lysine (left) and its acetylated form (right). (A) This perturbation topology is generated in the case when removing bonded interactions is allowed (improper dihedral angle around the nitrogen atom) and one of the following two options is used: either change in the multiplicity of dihedral angles is allowed or the procedure for matching dihedral angles uses only the middle atoms. (B) This definition of the perturbation path is obtained if the conditions from case A are not fulfilled or if perturbing bonds is not allowed. Note, however, that in this solution, the carbon atom of the acetyl group has five bonded interactions (one bond, two angles, one proper, and one improper dihedrals) with the unperturbed atoms, i.e., two redundant terms. Unperturbed atoms are highlighted in blue, perturbed atoms in red, and dummy atoms in gray.

Several additional options for adjusting the MCS search are available. Two different procedures for finding matches and mismatches between dihedral angles are implemented: (1) matching is allowed only if all (four) atoms of a dihedral angle in one topology match all atoms of a dihedral angle from the other topology and (2) a less restrictive procedure where matching is allowed if the middle two atoms match. The first option is preferred in the case when all possible dihedral angles are generated based on connectivity between atoms and the second option is favored if only one dihedral angle is defined per rotatable bond. In addition, the user can choose if the multiplicity of dihedral angles is allowed to be perturbed or not. Moreover, while the MCS search does not allow for creating or removing bonds, allowing such perturbations for other bonded interactions is optional. This primarily plays a role in the case of improper dihedral angles. Importantly, each of these options is independent of each other, permitting great flexibility in defining the rules for the MCS search. It is worth noting, however, that different combinations of choices might lead to the same solution, as illustrated in an example perturbation between lysine and *N*-acetyllysine (Figure 5).

Finally, as perturbation topologies inevitably involve noninteracting dummy atoms, additional care should be taken when handling such an atom. In particular, to ensure proper sampling of both states, dummy atoms should be attached to unperturbed atoms by three nonredundant bonded interactions.^64^ Additional bonded interactions (redundant terms) might affect the free-energy calculations and therefore should be removed. For example, the perturbation topology in Figure 5B would suffer from an such issue as the carbon atom of the acetyl group has five bonded interactions with the unperturbed atoms. As the removal of the redundant terms is complex and not uniquely defined,^64^ it is not implemented in the current version of the tool. However, upon detection of redundant terms, the user is prompted with a warning, which permits for manual curation of the proposed perturbation pathway.

### Multiple Topologies

**Figure 6:**
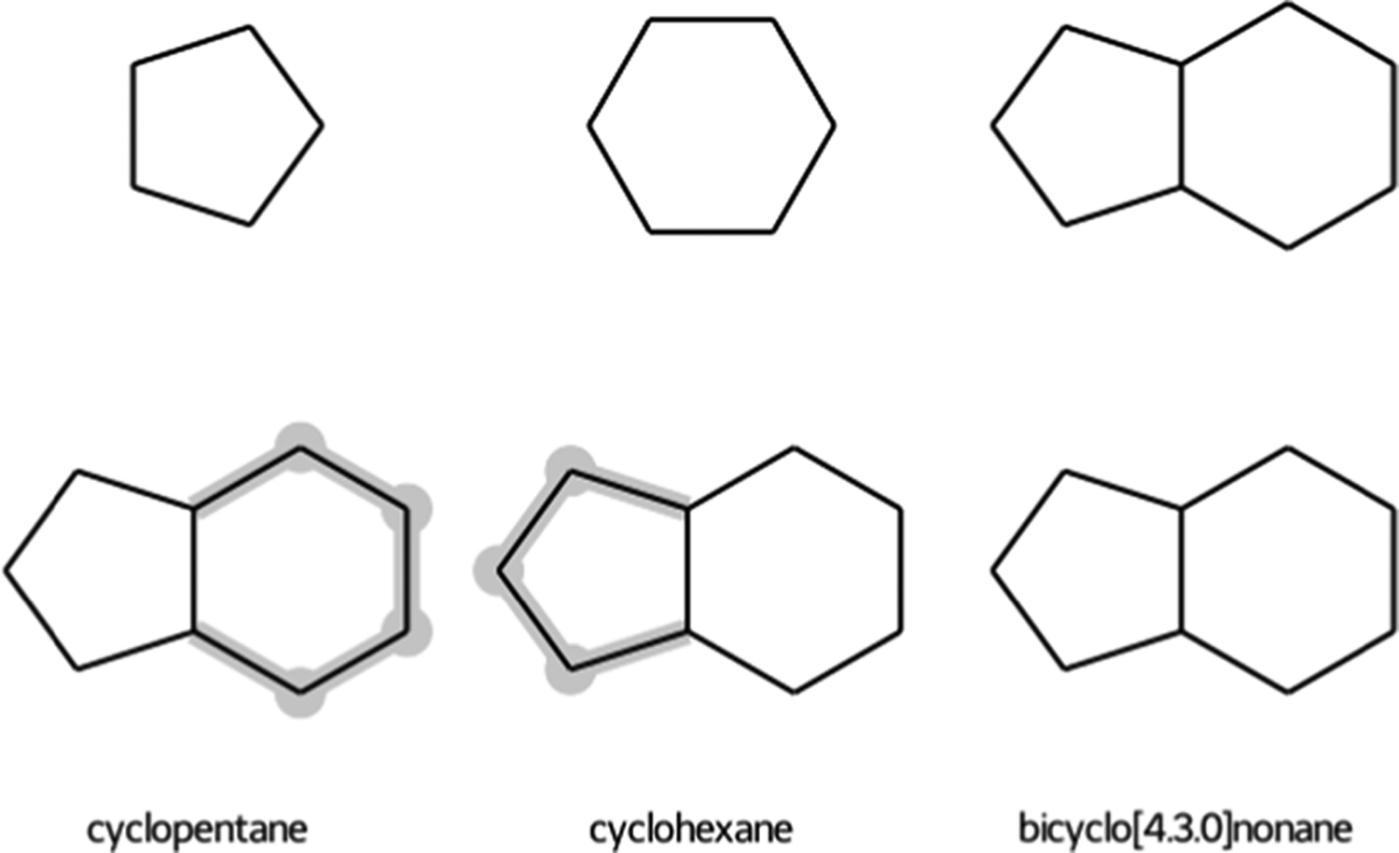
Multistate perturbation of three ring compounds. The top row represents individual compounds, while the bottom row corresponding EDS states where dummy atoms are highlighted in gray.

In addition to a pairwise (2 compounds) MCS search, it is possible to apply the algorithm on multiple compounds (three or more) simultaneously. This multistate search is primarily aimed at creating EDS topologies, where an EDS topology is a single topology (defining reference state Hamiltonian) that can represent multiple molecules by switching atom types, where the free-energy differences between the molecules are calculated using a one-step perturbation approach from the reference state. When applied on a set of simple compounds, including alkane chains and cycloalkanes of the same length with additional methyl groups at different positions (Figures S2 and S3 ) or polycycles (Figure 6), the algorithm is able to find the expected common substructures.

**Figure 7:**
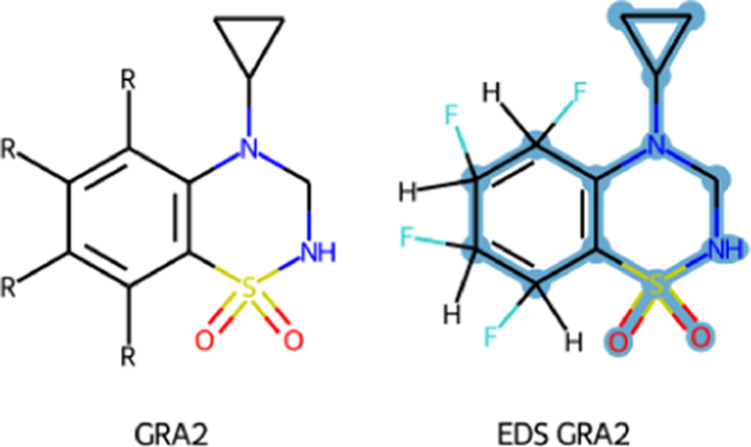
Different benzothiadiazine dioxide ligands of glutamate receptor A2 (GRA2), where different substituents (either a hydrogen or a fluoride atom) are represented with R (left). Multistate EDS topology representing all states, where the automatically recognized scaffold among the set of molecules (unperturbed atoms) is highlighted in blue, with one hydrogen and one fluoride atom attached to the common core to represent the substituents. A complete set of EDS states is shown in Figure S4.

In addition to these simplified test cases, in a recently published work,^43^ EDS topologies were generated using the default settings of the multistate algorithm, including a set of 16 glutamate receptor A2 (GRA2) allosteric modulators (Figure 7), a set of 8 trypsin inhibitors, and a set of 10 phenylethanolamine *N*-methyltransferase inhibitors. Note that the generation of the EDS topologies was done using the tools presented in this study, while the simulations and the data analysis are exclusively part of the reported publication.^43^

**Figure 8:**
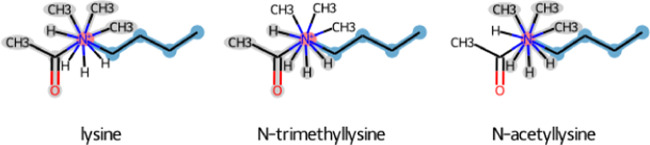
EDS topology representing lysine and two of its post-translational modifications. Unperturbed atoms are highlighted in blue, perturbed atoms in red, and dummy atoms in gray.

In addition to EDS topologies, such a multitopology approach can also be used to generate a closed thermodynamic cycle for a set of compounds. For example, when applied on a set of lysine post-translational modifications, a set of pairwise perturbation topologies between the states is obtained (Figure 8). These perturbation topologies, in addition to the related EDS topology, were used to calculate free-energy differences (see below).

Finding the maximum common substructure on a set of multiple topologies requires longer runtimes (in minutes), compared to pairwise topologies. Therefore, even though it is tempting to try applying this multitopology algorithm on a big set of diverse compounds, e.g., screening libraries of compounds, this would most probably lead to intractable running times. However, such sets of compounds are arguably also not relevant in the context of the EDS methodology or evaluation of the cycle closure since the number of states/perturbation legs would be too large for meaningful calculations. On the other hand, this toolkit offers an alternative way of tackling a large set of compounds by employing pairwise generation of perturbation topologies, potentially in combination with utilizing EDS techniques^41−43^ on small subgroups of similar compounds or by optimizing the choices of pairwise perturbations, as proposed by Liu et al.^65^

### Example Application of the Tool on a Set of Lysine Post-Translational Modifications

The application of the perturbation topology builder was illustrated on a simple test set containing lysine and two of its modified forms, including 3-methylation and acetylation modifications. The default multistate MCS search was performed, and perturbation topologies were generated (Figure 8) and used to calculate the free-energy differences between the states using the GROMOS and the GROMACS simulation packages (Table 1). Expectedly, the free-energy differences of lysine to *N*-trimethyllysine perturbation are practically indistinguishable between the two simulation packages, with 83.8 ± 0.1 and 83.6 ± 0.1 kJ mol^–1^ calculated using GROMOS and GROMACS, respectively. Interestingly, the other two perturbations involving a charge change result in slightly different free-energy differences. It is important to note that such perturbation calculations suffer from various artifacts, including one related with the choice of the cutoff scheme used in simulations (the DSM term in ref (66)). As different cutoff schemes were used when simulating with the two simulation packages (group-based and atom-based for GROMOS and GROMACS), contributing differently to the abovementioned artifact, the observed discrepancies in the free-energy differences are not surprising. This notwithstanding, the sum of the free-energy differences in the thermodynamic cycle of the three states is 0.5 ± 0.3 and 0.2 ± 0.2 kJ mol^–1^, obtained with the GROMOS and the GROMACS simulation packages, respectively. In addition to the pairwise perturbations, the related multistate topology was used in combination with the A-EDS to calculate the relative free energies between the states. The obtained results are in agreement with the pairwise free-energy calculations performed with the GROMOS simulation package (Table 1). This shows that the set of generated perturbation topologies is not only compatible between both simulation packages but also cross-method consistent.

**Table 1:** Free-Energy Differences between Lysine (LYS), *N*-Trimethyllysine (K3C), and *N*-Acetyllysine (KAC) Shown in kJ mol^–1 ^a

	LYS to K3C	K3C to KAC	KAC to LYS	sum
GROMACS	83.6 ± 0.1	69.5 ± 0.1	–153.3 ± 0.1	0.2 ± 0.2
GROMOS	83.8 ± 0.1	74.9 ± 0.1	–158.2 ± 0.2	0.5 ± 0.3
A-EDS (GROMOS)	83.4 ± 2.4	75.0 ± 0.6	–158.4 ± 2.5	

a Pairwise perturbations were performed using two different simulation packages, while accelerated EDS calculations were performed using the GROMOS simulation package.

## Conclusions

This study presents an automated tool for generating perturbation topologies (GROMOS and GROMACS file formats) based on the single topology approach by employing a maximum common substructure search algorithm. In each enumeration step of generating matched subgraphs, force-field-defined topology parameters are checked and stored. This allows for a flexible maximum common substructure search by setting a weighted preference toward minimizing perturbations of atom types or different types of bonded interactions such as bonds, angles, proper, and improper dihedrals. Any additional criteria may be added to guide the search, including the RMSD between the matched atoms in a set of coordinates. In addition to pairwise perturbation topologies between two states, the algorithm is able to generate a combined perturbation topology for a set of multiple topologies (three or more), which is primarily aimed to be used in combination with EDS techniques but can also be applied to define closed thermodynamic cycles.

Furthermore, the application of the generated perturbation topologies was illustrated by calculating the free-energy differences between lysine and two of its post-translational modifications, using the two simulation packages (GROMOS and GROMACS) and two approaches (pairwise transformations and the EDS method). Importantly, matching results were obtained (except for the perturbations involving net-charge change, most probably due to the differences in the cutoff schemes used) with almost ideal cycle closures, demonstrating that the perturbation topologies generated using this tool lead to a consistent set of alchemical transformations, readily used in the GROMOS and the GROMACS simulation packages.

Finally, it can be expected that the perturbation topology builder presented in this study, with automation, flexibility, and versatility in creating perturbation topologies, will improve the applicability of perturbation free-energy methodology in different contexts ranging from the estimation of protein stability to binding affinity calculations to rational drug development. To this end, this toolkit is provided as an open-source Python package via a GitHub repository (https://github.com/drazen-petrov/SMArt).

## Supporting Information Available

Illustration of the MCS when matching two ring structures for which no complete match was found, multistate perturbation topologies for a set of alkane chains, multistate perturbation topologies for a set of rings, and multistate perturbation topologies for a set of GRA2 allosteric modulators (PDF)

## Notes

The following software was used in this study: simulation packages GROMOS11, version 1.4.0 (http://www.gromos.net/) and GROMACS, version 2018.3 (http://www.gromacs.org/); pymbar implementation of the MBAR (http://github.com/choderalab/pymbar); and SMArt Python module available at (https://github.com/drazen-petrov/SMArt).

## Abbreviations

MD: molecular dynamics
MBAR: multistate Bennett acceptance ratio
MCS: maximum common substructure
GRA2: glutamate receptor A2
