# Supplementary material for: Perturbation Free-Energy Toolkit: An Automated Alchemical Topology Builder
Source: J Chem Inf Model. 2021 Aug 20;61(9):4382–90. doi: 10.1021/acs.jcim.1c00428 (PMC8479811; doi:10.1021/acs.jcim.1c00428)
Supplement: Supplementary file 1 — ci1c00428_si_001.pdf [file ci1c00428_si_001.pdf]

Supporting Information

Perturbation Free-Energy Toolkit: Automated Alchemical Topology Builder

Drazen Petrov[1*]

[1]Department of Material Sciences and Process Engineering, Institute of Molecular Modeling and Simulation, University of Natural Resources and Life Sciences Vienna, Muthgasse 18, A-1190 Vienna, Austria

*corresponding author

SUPPORTING INFORMATION TABLES AND FIGURES

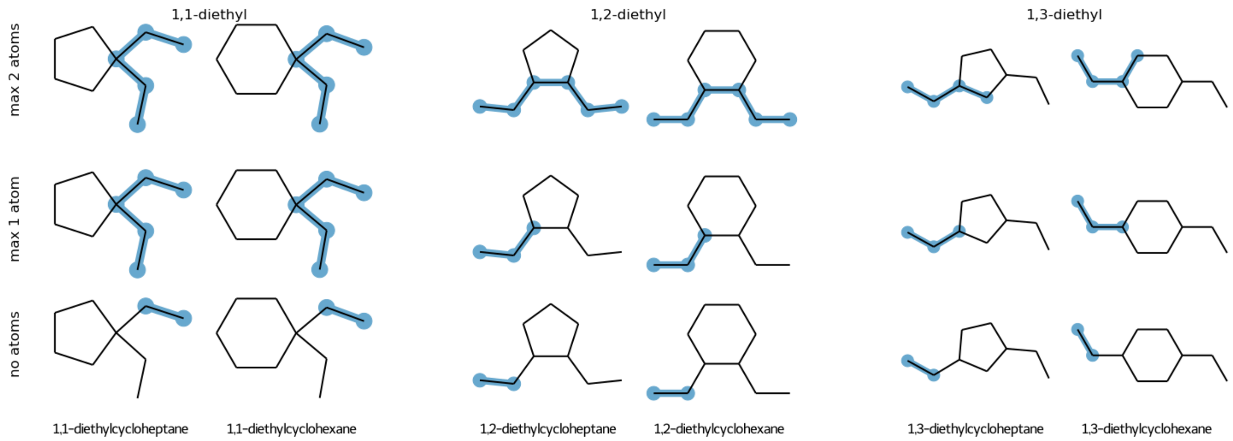

Figure S1. Illustration of the maximum common substructure (highlighted in blue) when matching two ring structures for which no complete match was found. Top row: partial match of maximum of 2 atoms (that share a bond); middle row: partial match of only one atom; and bottom row: no atom match allowed. For simplicity, only the MCS is shown without perturbation topologies that contain additional dummy atoms that are not part of the MCS match.

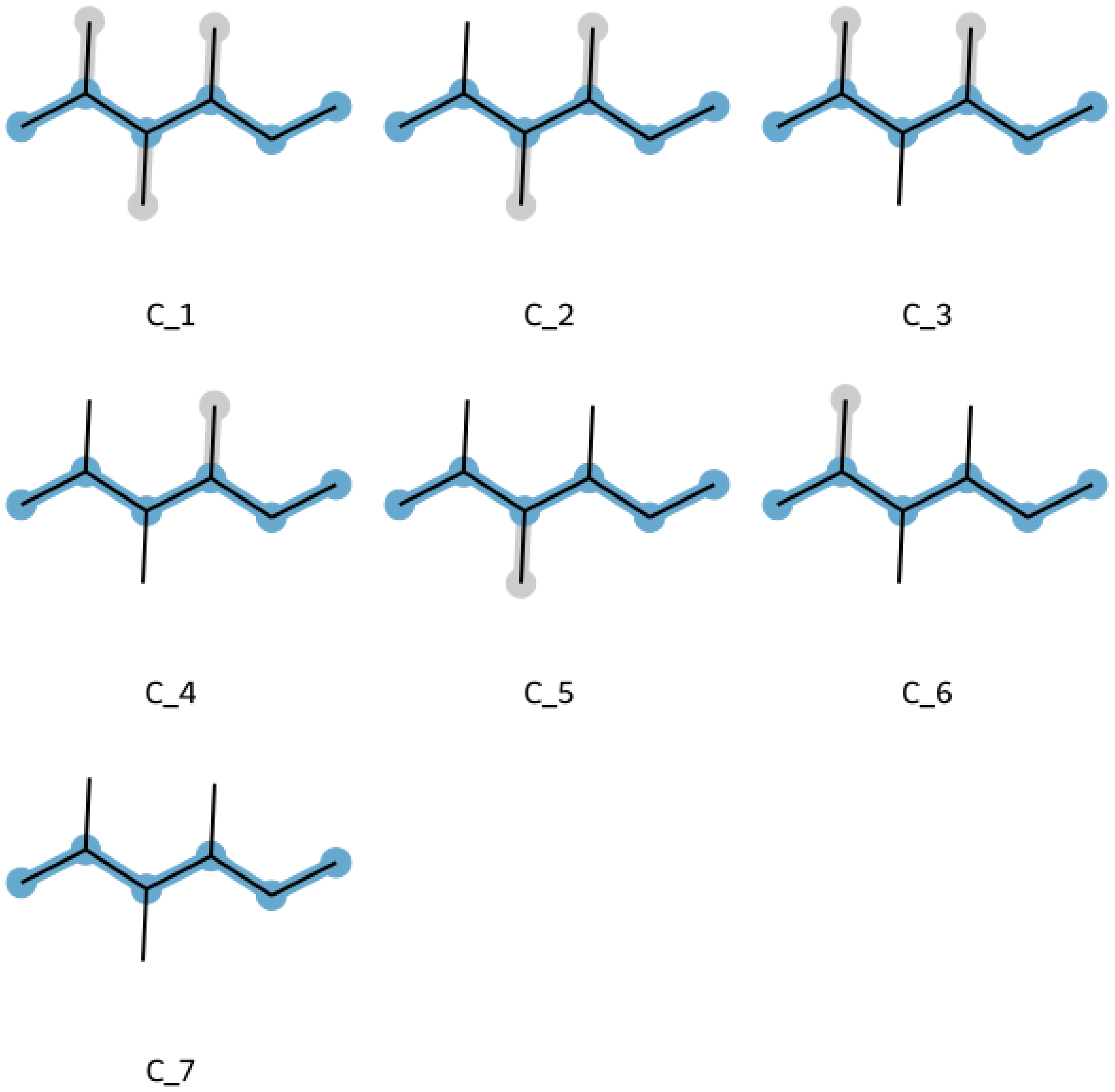

Figure S2. Multi-state perturbation solution for a set of alkane chains. The unperturbed atoms (common core) are highlighted in blue, while non-interacting dummy are highlighted in gray.

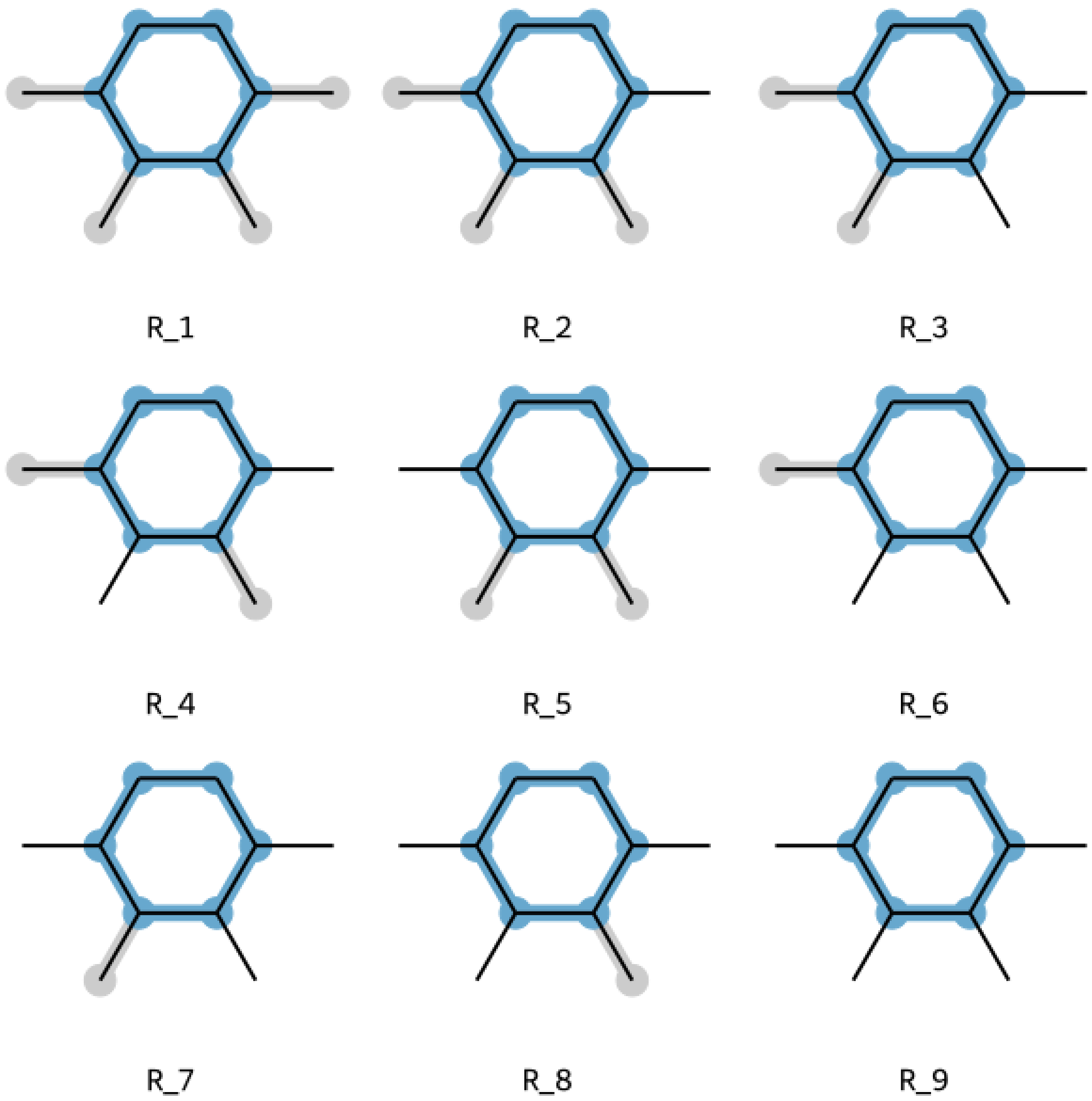

Figure S3. Multi-state perturbation solution for a set of rings. The unperturbed atoms (common core) are highlighted in blue, while non-interacting dummy are highlighted in gray.

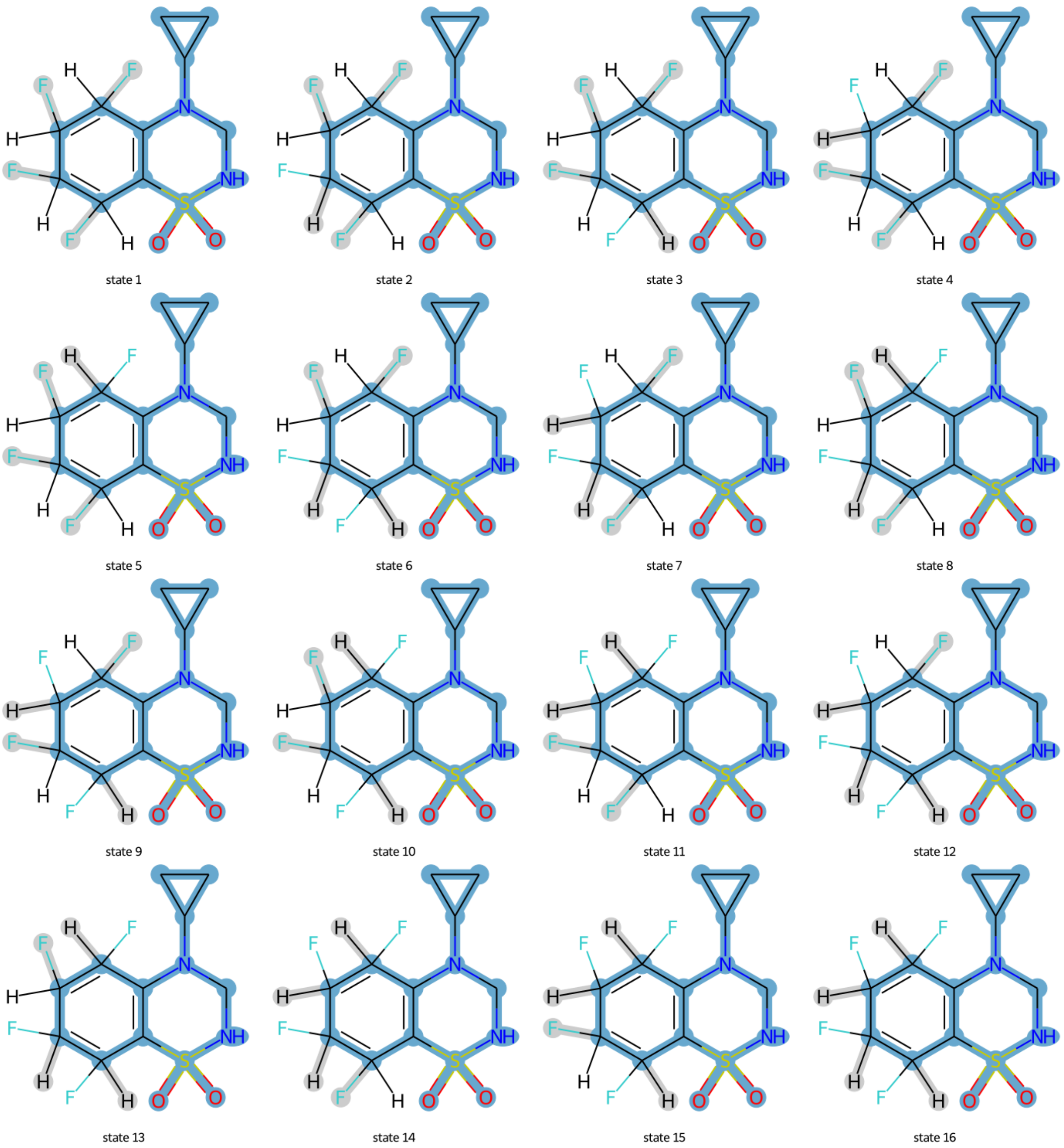

Figure S4. A complete set of 16 EDS states of benzothiadiazine dioxide ligands of glutamate receptor A2 (GRA2). Unperturbed atoms are highlighted in blue, perturbed atoms in red, and dummy atoms in gray.
